# The global, regional, and national burden of gastroesophageal reflux disease in 204 countries and territories, 1990–2021: a retrospective cohort study

**DOI:** 10.1097/MS9.0000000000003493

**Published:** 2026-03-18

**Authors:** Lichuan Du, Yan Gao, Ru Gao

**Affiliations:** Gastroenterology Department, Beijing Chaoyang Hospital, Capital Medical University, Beijing, China

**Keywords:** age-standardized rate, epidemiology, gastroesophageal reflux disease, global burden disease

## Abstract

**Introduction:**

Gastroesophageal reflux disease (GERD) is a common condition involving recurrent reflux of stomach contents, causing symptoms and complications that impact quality of life. This study aimed to assess the global GERD burden from 1990 to 2021.

**Methods:**

We used the Global Burden of Disease 2021 data to analyze the incidence, prevalence, and years lived with disability (YLDs) for GERD across 204 countries. We estimated the population-level distributions by age and sex, using age-standardized incidence rate (ASIR) and age-standardized YLDs (ASYLDs). The average annual percent change (AAPC) in incidence, prevalence, and YLDs was calculated along with 95% uncertainty intervals (UIs).

**Results:**

In 2021, the global ASIR of GERD was 3881.86 per 100 000, reflecting an AAPC increase of 3.80% since 1990. The prevalence also increased, with an AAPC of 3.38%, reaching 9838.60 per 100 000. Global ASYLDs rose to 75.56 per 100 000, showing an AAPC increase of 3.49%. GERD burden varied by region; high-income areas like North America and East Asia saw declines, while Western Sub-Saharan Africa and Central Europe showed increases. India and China had the highest recorded incidences, with 36 567 410 and 20 863 747, respectively. Decomposition analyses revealed that population growth and aging contributed most to the increase in YLDs.

**Conclusion:**

The global GERD burden significantly increased from 1990 to 2021, especially in low and middle-income regions. This highlights the urgent need for enhanced public health measures, early diagnosis, and improved healthcare access to manage the growing disease burden and improve patient outcomes.

## Introduction

Gastroesophageal reflux disease (GERD) is a chronic digestive disorder that affects approximately 20% of adults in Western countries, with varying prevalence across different regions globally^[^1^]^. GERD is characterized by recurrent reflux of stomach contents into the esophagus, leading to symptoms such as heartburn and acid regurgitation^[^2,3^]^. Beyond causing discomfort, GERD is associated with serious complications, including Barrett’s esophagus and esophageal adenocarcinoma, thereby representing a significant public health issue^[^4–6^]^. Over the past few decades, the global incidence and prevalence of GERD have been increasing, affecting individuals of all age groups and across diverse regions^[^7^]^. Despite GERD’s substantial impact, comprehensive data on its global, regional, and national burden remain limited. Most existing studies focus on specific countries or populations, creating gaps in understanding the overall scope of the disease worldwide^[^8–10^]^. This lack of detailed epidemiological data limits the development of effective preventive strategies and the efficient allocation of healthcare resources to manage GERD. By investigating the trends and demographic factors influencing GERD, this study aims to provide a foundation for targeted interventions and policy development to mitigate its impact.

Recent systematic reviews and studies have established that GERD is not only a prevalent health issue but also a precursor to more severe esophageal conditions^[^11,12^]^. For instance, the association between long-standing GERD and the risk of esophageal cancer underscores the need for a comprehensive understanding of its global epidemiology and the efficacy of current management strategies^[^13^]^. Understanding the progression from GERD to complications such as Barrett’s esophagus is critical for the development of early intervention and prevention strategies. Additionally, regional and population-based variations in GERD prevalence are likely influenced by factors such as dietary habits, obesity, and *Helicobacter pylori* infection rates, necessitating further research^[^14^]^. Through this study, we aim to elucidate the shifts in GERD’s global burden and identify potential underlying causes to better inform future healthcare strategies.

The Global Burden of Disease (GBD) 2021 Study, one of the most extensive worldwide observational epidemiological studies to date, assesses the incidence, prevalence, and disability associated with various diseases by age, sex, location, and year(“Global Burden of Disease Study 2021 (GBD 2021) Data Resources|GHDx,” n.d.). This provides an opportunity to enhance our understanding of GERD epidemiology. In this article, we aim to describe the burden of GERD based on age, sex, and sociodemographic index (SDI) in 204 countries and territories from 1990 to 2021. The availability of comprehensive estimates of GERD burden will contribute to a deeper understanding of its impact on population health and highlight the need for appropriate preventive strategies and healthcare resource allocation.

## Methods

### Data sources

This study utilizes data from the GBD 2021, which compiles comprehensive global health statistics including incidence, prevalence, and years lived with disability (YLDs) for GERD from 1990 to 2021 across 204 countries and territories. The GBD data set, derived from a variety of primary and secondary data sources, provides a rigorous and peer-reviewed methodology ensuring high validity and reliability of the estimates.

### Study design

The study was designed as a retrospective analysis using the systematic analytical framework provided by the GBD study. We assessed the age-standardized incidence rates (ASIR), age-standardized prevalence rates, and age-standardized years lived with disability for GERD. These indicators help standardize the measurement across different populations, adjusting for age variations and enabling comparative assessments over time and between regions.

### Statistical analysis

Statistical analysis was performed using the metrics of age-standardized rates (ASRs) provided by the GBD 2021 study. For temporal trend analysis, we calculated the average annual percent change in incidence, prevalence, and YLDs using a log-linear model. Confidence intervals (95% CI) and uncertainty intervals (UI) were also derived to quantify the precision of the estimates. The decomposed analysis was utilized to separate the contributions of population growth, aging, and disease prevalence to the changing burden of GERD. Additionally, frontiers analysis was conducted to assess the potential for improvement based on the SDI.

As this study did not involve any human participants, patient data, or interventions, ethical approval was not required. The study utilized publicly available data from the GBD 2021, which does not involve identifiable personal information or require specific ethical oversight. This study adheres to the Strengthening the Reporting of Cohort Studies in Surgery guidelines^[^15^]^ to ensure comprehensive and transparent reporting of observational research.

Data analyses and visualization were performed between July to September 2024 using R software (version 4.2.1). All datasets were downloaded from the publicly available Global Health Data Exchange (GHDx) portal. As the GBD dataset is open-access and fully anonymized, no ethical approval was required. Data integrity was ensured through standardized data cleaning procedures and version tracking using Git. Both raw and processed data were securely stored in a hospital-based medical research repository compliant with institutional data governance policies.

**HIGHLIGHTS**

- Global increase in gastroesophageal reflux disease (GERD) burden: From 1990 to 2021, age-standardized incidence, prevalence, and years lived with disability (YLDs) for GERD rose by 3.80%, 3.38%, and 3.49%, respectively, driven by population growth and aging.
- Divergent regional trends: High-income regions (e.g. North America, East Asia) saw declines in GERD burden, while low/middle-income regions (e.g. Western Sub-Saharan Africa, Central Europe) experienced rising incidence, prevalence, and YLDs.
- Country-specific insights: India and China reported the highest absolute incident cases (36.6 million and 20.9 million, respectively), despite reductions in age-standardized rates.
- Socio-demographic disparities: Lower sociodemographic index (SDI) regions faced higher mortality and disability-adjusted life years, reflecting gaps in healthcare access and diagnostic capacity.
- Age and sex patterns: GERD burden peaked in middle-aged adults (45–49 years) and elderly populations (65–74 years), with males consistently exhibiting higher rates than females.
- Policy implications: Urgent need for region-specific interventions, including enhanced primary care integration in low-SDI regions and strategies to reduce overtreatment in high-SDI settings.

## Results

### Global burden and temporal trend in GERD

According to global data on GERD, there were 324.1 million incident cases worldwide (95% UI: 287.7–358.9 million), corresponding to an ASR of 3881.86 per 100 000 population, reflecting a 3.80% increase compared to 1990. The global prevalence of GERD reached 825.6 million cases (95% UI: 732.99–925.55 million), with an ASR of 9838.60 per 100 000 population, reflecting a 3.38% increase since 1990. YLDs totaled 6.34 million (95% UI: 3.19–11.24 million), with an ASR of 75.56, indicating a 3.49% increase (Table 1).

**Table 1:** Incident cases, prevalent cases, and YLDs of gastroesophageal reflux disease in 2021 and percentage change from 1990 in age-standardized rates per 100 000 population for both sexes by GBD regions.

Region	Inc_ASR	Inc_Change	Prev_ASR	Prev_Change	YLD_ASR	YLD_Change
Global	3881.86 (3445.56, 4299.95)	3.80 (3.51, 4.21)	9838.60 (8732.46, 11056.05)	3.38 (3.21, 4.11)	75.56 (38.05, 133.87)	3.49 (1.79, 5.76)
Southeast Asia	2222.39 (1955.85, 2511.31)	−0.04 (−0.04, −0.05)	5493.35 (4809.00, 6212.23)	−0.03 (−0.04, 0.02)	42.37 (21.29, 76.29)	0.10 (0.01, 0.33)
Oceania	2167.33 (1897.19, 2443.04)	0.03 (0.01, 0.04)	5325.80 (4658.08, 6017.80)	0.03 (0.02, 0.04)	40.87 (20.80, 73.02)	0.04 (0.05, −1.38)
East Asia	1849.87 (1609.66, 2090.47)	−0.34 (−0.63, −0.19)	4554.18 (3961.91, 5170.75)	−0.40 (−0.46, −0.39)	35.22 (17.68, 62.55)	−0.35 (−0.10, −1.01)
Southern Sub-Saharan Africa	4663.54 (4108.83, 5192.32)	0.07 (0.08, 0.10)	11804.55 (10447.92, 13226.90)	0.10 (0.11, 0.12)	89.67 (45.30, 160.39)	−0.66 (−0.26, −1.34)
Eastern Sub-Saharan Africa	4583.75 (4027.78, 5100.72)	0.08 (0.05, 0.10)	11595.51 (10229.70, 13050.55)	0.09 (0.06, 0.06)	88.84 (44.90, 159.47)	0.41 (0.14, 0.54)
Andean Latin America	6099.59 (5437.15, 6734.20)	−0.04 (−0.02, −0.06)	16405.98 (14495.47, 18306.62)	−0.02 (−0.02, −0.05)	126.58 (64.01, 223.80)	−0.12 (0.00, −0.31)
Central Europe	4392.32 (3870.96, 4864.31)	0.66 (0.65, 0.73)	11189.98 (9850.75, 12545.83)	0.79 (0.63, 0.86)	86.05 (43.35, 152.64)	1.07 (0.42, 2.02)
Western Europe	3296.77 (2878.87, 3692.37)	−0.04 (0.12, 0.36)	8222.18 (7208.31, 9249.61)	−0.13 (−0.55, 0.05)	63.30 (31.76, 112.67)	−0.07 (−0.18, −0.49)
High-income North America	3794.49 (3313.53, 4242.52)	−7.70 (−6.68, −7.89)	9595.09 (8402.41, 10842.16)	−10.25 (−8.21, −11.35)	73.21 (37.26, 131.48)	−10.81 (−4.95, −18.63)

### Regional analysis

In 2021, high-income regions like North America and East Asia are experiencing significant declines in GERD incidence and prevalence, while regions such as Western Sub-Saharan Africa and Central Europe are witnessing increases across three indicators.

**Figure 1.**
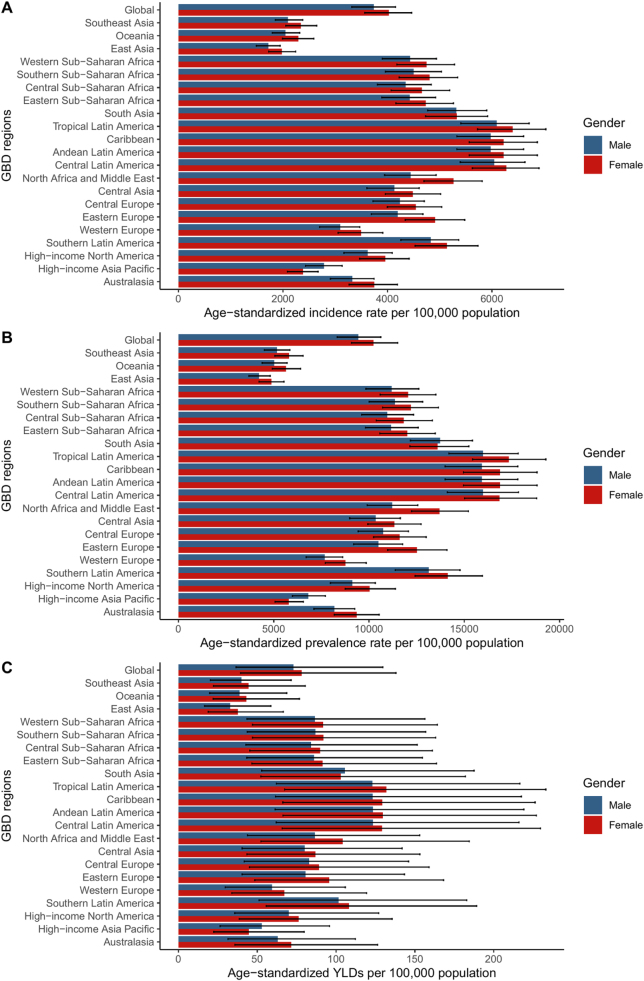
The age-standardized incidence (A), age-standardized prevalence (B), and YLDs (C) of GERD for GBD regions by sex, 2021. GBD, global burden of diseases, injuries, and risk factors study.

Southeast Asia reported 16.34 million incident cases (ASR 2222.39), a −0.04% change since 1990. East Asia showed 33.64 million incident cases (ASR 1849.87), a −0.34% change. Western Sub-Saharan Africa had 15.64 million incident cases (ASR 4601.02), reflecting a 0.11% increase. High-income North America reported 17.49 million incident cases (ASR 3794.49), indicating a −7.70% reduction (Fig. 1, Table 1).

**Figure 2.**
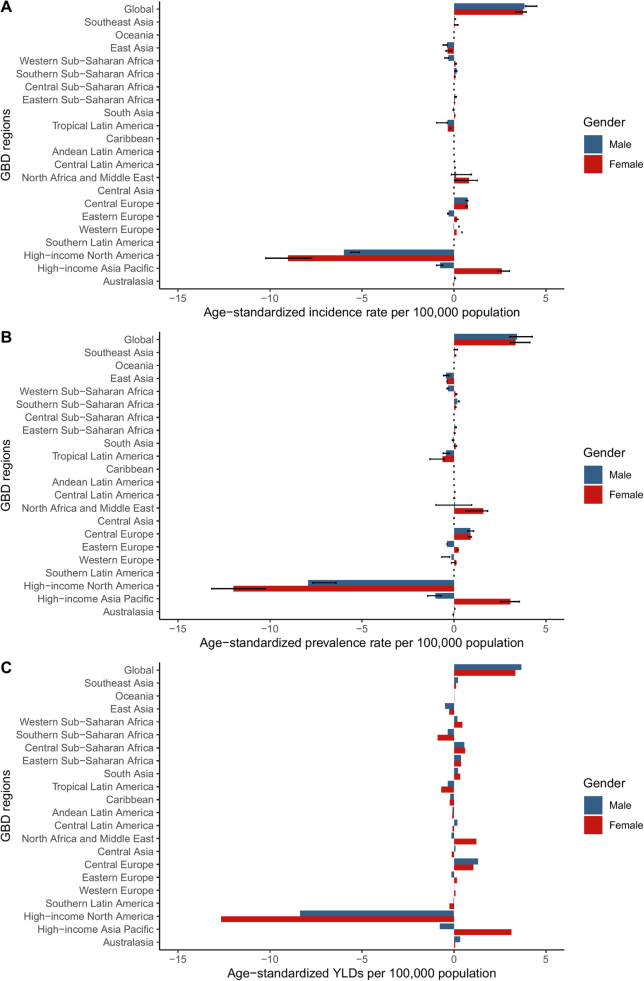
The percentage change in age-standardized incidence (A) age-standardized prevalence (B) and YLDs (C) of GERD for GBD regions by sex, 1990−2021. GBD, global burden of diseases, injuries, and risk factors study.

Several regions, such as Central Europe and Western Sub-Saharan Africa, showed increasing trends in incidence, prevalence, and YLDs. Other areas, such as Australasia and Southern Latin America, remained stable. In Asia, East Asia experienced a reduction across all metrics, while South Asia showed relatively minor changes (Fig. 2).

From 1990 to 2021, GERD burden slightly increased globally, with consistent declines observed in high-income regions and upward trends in Sub-Saharan Africa and Central Europe (Fig. 2).

### National and territorial trends in GERD from 1990 to 2021

In 1990, India reported 76 322 880 incident cases of GERD, which declined to 36 567 410 in 2021. China’s incidence also decreased from 32 387 866 in 1990 to 20 863 747 in 2021. Similar downward trends were observed in other countries such as the United States, Brazil, and Russia. Despite reductions in total case numbers, the ASR showed minimal change in many countries, suggesting demographic shifts.

**Figure 3.**
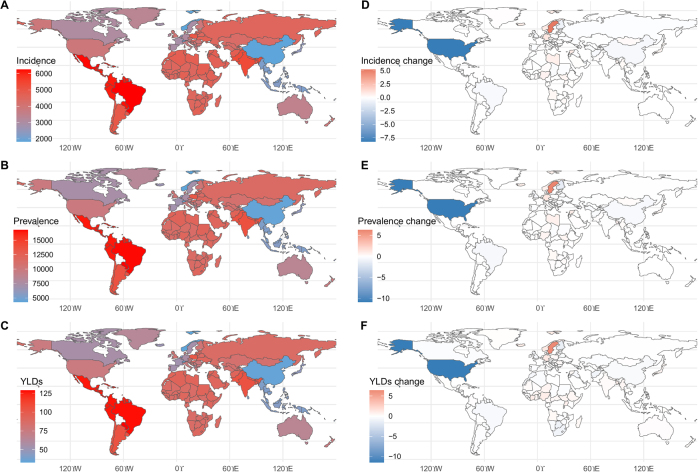
The age-standardized rates and percentage change in age-standardized rates of GERD for 204 countries and territories. (A) The age-standardized incidence rates of GERD, 2021. (B) The age-standardized mortality rates of GERD, 2021. (C) The age-standardized DALY rates of GERD, 2021. (D) The percentage change in age-standardized incidence rates of GERD, 1990–2021. (E) The percentage change in age-standardized mortality rates of GERD, 1990–2021. (F) The percentage change in age-standardized DALY rates of GERD 1990–2021. DALY, disability-adjusted life-year.

For example, in India, GERD prevalence declined from approximately 825 million in 1990 to 451 million in 2021. This was accompanied by only slight changes in ASR, indicating that changes in raw numbers may be due in part to population structure rather than epidemiologic improvements. Across 204 countries and territories, the percent change in ASR for incidence and prevalence ranged from modest to substantial declines (Fig. 3).

In Afghanistan, GERD incidence in 2021 was 356 327 cases (ASR: 4827.00 per 100 000), compared to 1 019 396 cases (ASR: 4809.18) in 1990. The DALY count increased from 19 046 to 6685 during the same period, with ASR changing from 92.70 to 93.47 per 100 000 population.

### Burden of GERD by SDI (1990–2021)

Data from 1990 to 2021 show that regions with higher SDI exhibited higher GERD incidence rates but lower mortality and DALY rates. For example, High-income North America, Western Europe, and Australasia reported incidence rates exceeding 5000 per 100 000 population. In contrast, Sub-Saharan Africa and South Asia generally had incidence rates below 3000 per 100 000.

**Figure 4.**
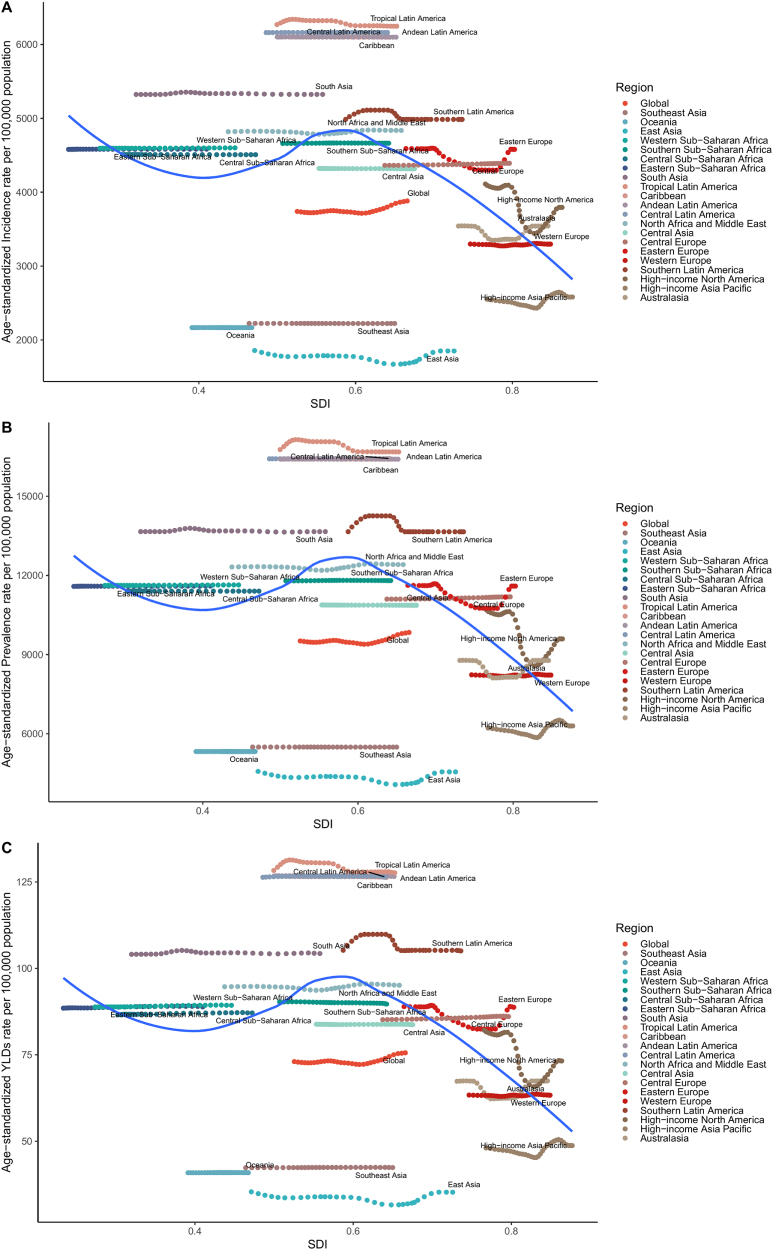
Age-Standardized Rates of GERD by SDI (1990–2021). (A) Age-standardized incidence rates per 100 000 population by SDI. (B) Age-standardized mortality rates per 100 000 population by SDI. (C) Age-standardized DALY rates per 100 000 population by SDI.

**Figure 5.**
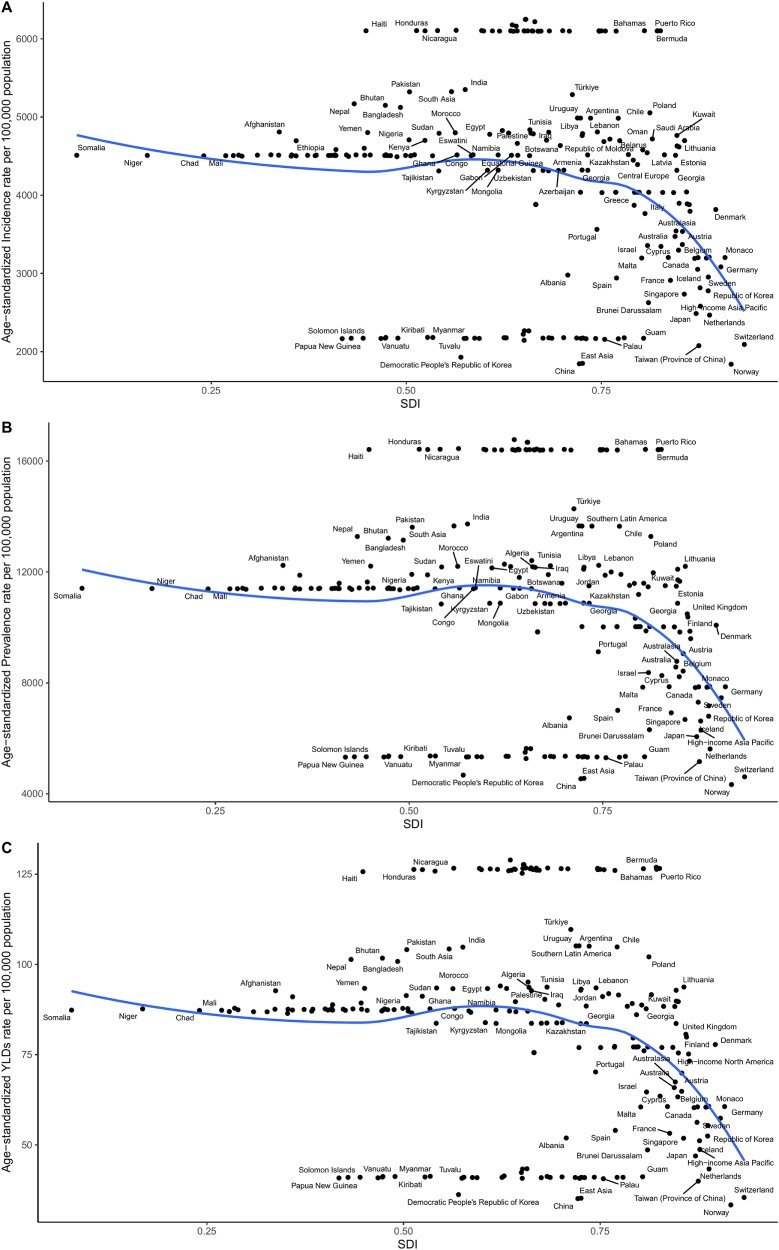
National burden of GERD by SDI (1990–2021). (A) Age-standardized incidence rates per 100 000 population across countries by SDI. (B) Age-standardized mortality rates per 100 000 population across countries by SDI. (C) Age-standardized DALY rates per 100 000 population across countries by SDI.

Age-standardized mortality rates decreased with increasing SDI. In High-income North America, mortality rates were well below 1000 per 100 000 population, whereas countries such as Somalia and Niger exhibited higher mortality burdens (Figs. 4B and 5B).

DALY rates followed similar patterns: high-SDI regions had lower age-standardized DALY rates, while lower-SDI countries such as those in Sub-Saharan Africa maintained higher DALY burdens, reflecting persistent gaps in healthcare access and disease management (Figs. 4C and 5C).

### Age and sex-related patterns in the global burden of GERD in 2021

In 2021, the highest number of GERD incident cases occurred in the 45–49 age group for both males and females. After this peak, the number of incident cases declined with age. ASIR also peaked in this group, then declined slightly but remained elevated into older age.

GERD-related mortality peaked in the 65–69 age group for both sexes, with males showing higher mortality across nearly all age groups. The age-standardized mortality rate continued to increase with age, reaching its highest point in populations over 80 years old. The UIs widened in older age brackets.

**Figure 6.**
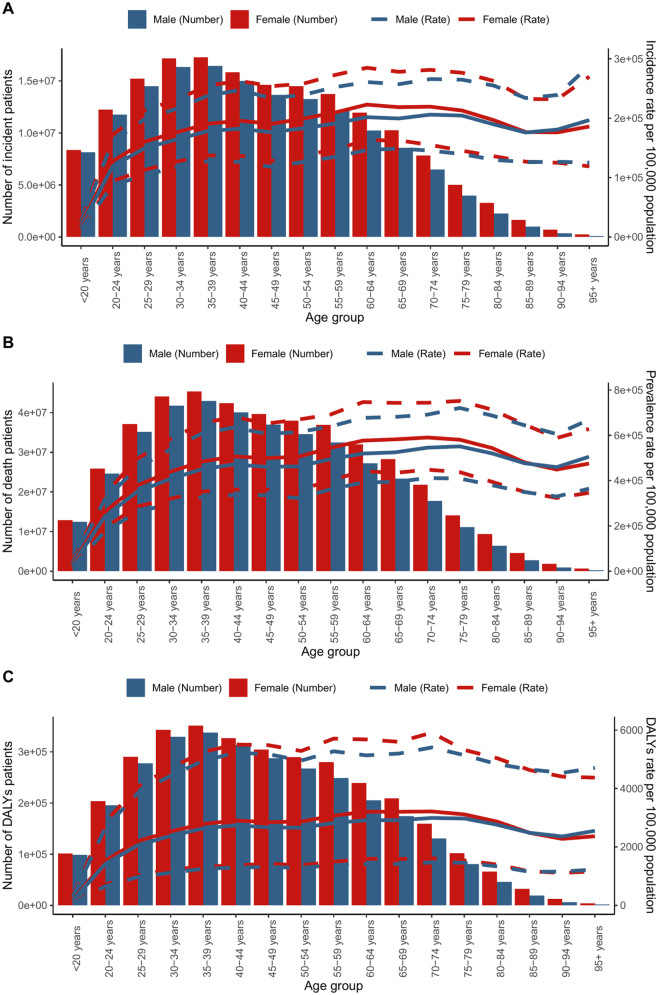
Global number and age-standardized rates of incidence (A), deaths (B), and DALYs (C) of GERD by age and sex, 2021. The box plots indicate numbers and line plots indicate rates per 100 000 population. Dashed lines indicate 95% upper and lower uncertainty intervals. DALY, disability-adjusted life-year.

Disability-adjusted life years (DALYs) peaked between ages 70–74, with males consistently reporting higher DALY counts than females. While DALY rates declined slightly beyond the 75+ age group, they remained high among elderly populations, indicating a significant disease burden in later life (Fig. 6).

## Discussion

### Main findings

This study provides one of the most comprehensive assessments to date of the global, regional, and national burden of GERD, drawing from data in 204 countries and territories over three decades. Our findings reveal a global increase in GERD incidence, prevalence, and YLDs between 1990 and 2021. Despite relatively stable ASRs in many regions, the absolute number of cases has risen sharply due to population growth and aging. Substantial variation was observed across SDI quintiles, geographic regions, age groups, and sex. These findings reflect not only epidemiological transitions, but also underlying inequities in healthcare access, disease awareness, and risk factor exposure.

### Global trends and regional disparities in GERD burden

Our results show that high-income regions such as North America and East Asia experienced notable declines in GERD ASRs, while low- and middle-income regions, including Western Sub-Saharan Africa and Central Europe, witnessed increasing burdens. For instance, Western Sub-Saharan Africa showed a 0.53% increase in YLDs, while Central Europe experienced a 1.07% increase. These patterns align with global transitions in diet, obesity, and healthcare infrastructure. In contrast to earlier studies focusing on high-income countries^[^1,14,16^]^, we reveal a shift in disease concentration toward populous and emerging economies. India and China, despite reporting reductions in ASR, still accounted for the highest absolute number of incident cases in 2021 (36.5 and 20.9 million, respectively). These trends may reflect rapid demographic expansion coupled with health system strain, where improvements in early detection have not kept pace with epidemiological pressure. The sharp decline in high-income North America (−10.81% in YLDs) likely reflects a combination of established screening systems, widespread use of proton pump inhibitors (PPIs), public health messaging, and possibly shifts in *H. pylori* prevalence and other risk exposures^[^17,18^]^. However, even in these settings, concerns around overtreatment, long-term PPI use, and under-recognized extraesophageal manifestations of GERD^[^2,3^]^ persist, suggesting that declining ASRs may not fully reflect the lived burden of disease.

### Socio-demographic inequities: SDI as a structural determinant

The inverse gradient observed between SDI and GERD-related DALYs suggests that disease burden is not solely driven by biological or behavioral factors, but by structural determinants. In our study, high-SDI regions reported higher incidence but lower mortality and DALYs, while low-SDI regions demonstrated the opposite. This reflects both improved detection capacity in developed settings and greater functional burden in under-resourced systems. These observations are consistent with previous studies that have pointed to an emerging GERD burden in lower-income regions such as Sub-Saharan Africa and Central Europe^[^9,10,19^]^. For instance, our findings reveal that Western Sub-Saharan Africa experienced increases across all burden indicators from 1990 to 2021, including a 0.53% rise in YLDs and a 0.15% increase in prevalence ASR. Similarly, Central Europe reported a 0.66% increase in ASR for incidence and a 1.07% increase in YLDs. These region-specific trends extend earlier research by quantifying not only GERD presence, but the disability it causes in health systems already stretched by epidemiological transitions^[^9^]^. Afghanistan offers a further illustrative example: while its incidence ASR remained nearly unchanged between 1990 and 2021, DALY ASR increased slightly (from 92.70 to 93.47), indicating growing disease impact relative to stable detection. Such discrepancies may result from limited availability of diagnostic tools, delayed care-seeking, and low awareness about atypical GERD symptoms. Together, these findings reinforce the view that SDI serves as more than a descriptive metric, it operates as a structural determinant of health equity, access, and long-term outcomes. Countries with lower SDI often face a dual burden: rising disease prevalence combined with limited system responsiveness to manage and mitigate chronic illness^[^20,21^]^.

### Demographic patterns: intersections of age, sex, and physiology

Our age- and sex-disaggregated analysis revealed that GERD burden peaks in the 45–49 and 65–74 age groups for incidence and DALYs, respectively, and is consistently higher among males. While this aligns with earlier findings^[^22–24^]^, our data clarify how these patterns vary by region. For example, male predominance was consistent across both low- and high-SDI countries, suggesting a combination of biological and behavioral drivers.

Age-related vulnerability may be linked to decreased esophageal clearance, weakened lower esophageal sphincter tone, and comorbidities that amplify symptom severity^[^22^]^. In low-resource settings, older adults may face delayed care-seeking or financial barriers, compounding their risk. Additionally, the use of medications that exacerbate reflux (e.g. calcium channel blockers, sedatives) is higher among older populations, potentially increasing the physiological burden of disease.

The consistent sex disparity observed, with males exhibiting higher ASRs, may reflect differences in visceral fat distribution, hormonal protection among premenopausal women, and lifestyle-related exposures (e.g. alcohol, tobacco)^[^23^]^. However, gendered norms and biases in diagnosis and health-seeking behavior may also contribute, especially in societies where women underreport symptoms or prioritize family health over personal care^[^25^]^.

### Strengths and limitations

This study is among the most comprehensive assessments of GERD burden worldwide, using standardized GBD data across 204 countries. However, limitations include potential underreporting in low-income settings, the use of modeled rather than raw data, and limited granularity in capturing individual-level risk factors such as dietary patterns and medication use.

### Policy implications and future directions

The decomposition analysis confirms that population growth and aging are primary contributors to the global increase in GERD burden. This has immediate implications for health policy, particularly in countries undergoing demographic transition without corresponding health system development. To address this burden, high-SDI regions should prioritize reducing overmedicalization, promoting rational use of long-term pharmacological treatments such as PPIs, and enhancing patient education regarding modifiable risk factors. In contrast, low- and middle-SDI countries require foundational investments, such as integrating GERD screening and management into primary care, adopting task-shifting strategies to empower nonspecialist providers, and launching culturally tailored symptom awareness campaigns to improve early detection and patient engagement.

Future studies should explore the socio-behavioral and environmental drivers of GERD, especially in lower-SDI regions where data are often sparse and the disease remains underdiagnosed. Improving diagnostic access and strengthening public health literacy, particularly regarding dietary triggers, sleep hygiene, and healthcare-seeking behavior are critical areas for targeted intervention. Moreover, global GERD strategies must account for its frequent comorbidities, including obesity, obstructive sleep apnea, anxiety, and depression, which may intensify disease burden and complicate treatment outcomes. Longitudinal, multicenter studies linking GERD with lifestyle and psychological factors in diverse populations, particularly in non-Western and underserved settings are essential to develop equitable, evidence-based prevention and management strategies.

## Conclusion

GERD continues to pose a significant public health challenge, with a rising burden globally from 1990 to 2021. Despite observed decreases in high-income regions, the burden remains substantial in lower-income areas, reflecting disparities in healthcare access, diagnostic capabilities, and treatment. Effective management of GERD requires a combination of public health initiatives focused on lifestyle modifications, improved diagnostic procedures, and enhanced access to medical treatment. Future strategies should emphasize early diagnosis, preventive measures, and region-specific interventions to mitigate the increasing trend of GERD globally. Strengthening healthcare infrastructure in low and middle-income countries and developing tailored interventions are essential steps to alleviate the growing burden in emerging hotspots.

## Data Availability

The datasets generated during and/or analyzed during the current study are publicly available from the Global Cancer Observatory (https://gco.iarc.fr/) and the Global Burden of Disease study (https://www.healthdata.org/gbd).

## Research registration unique identifying number (UIN)

This study utilized publicly available data and does not involve new human subjects. Registration was not applicable.
